# Limitations of repurposing ceftazidime for *Stenotrophomonas maltophilia*: a cautionary perspective

**DOI:** 10.1128/msphere.00384-25

**Published:** 2025-08-04

**Authors:** Madhumathi Irulappan, Balaji Veeraraghavan

**Affiliations:** 1 Department of Clinical Microbiology, Christian Medical College 196380, Vellore, Tamil Nadu, India; University of Nebraska Medical Center College of Medicine, Omaha, Nebraska, USA

**Keywords:** *Stenotrophomonas maltophilia*, RPMI-1640 vs CAMHB, polymicrobial infections, pharmacokinetics, media-dependent susceptibility

## LETTER

We read with interest the recent study by Phillips et al., exploring ceftazidime (CAZ) repurposing for *Stenotrophomonas maltophilia* based on improved activity in nutrient-depleted RPMI-1640 medium (1). By challenging the conventional use of cation-adjusted Mueller-Hinton broth (CAMHB) for susceptibility testing, the study adds to ongoing discussions about the relevance of *in vitro* conditions in predicting clinical efficacy, especially amid growing multidrug resistance.

Though exploratory, the study underscores a critical issue: standard *in vitro* methods may not always reflect *in vivo* outcomes. This has broader implications for cephalosporins, many of which have seen reduced clinical use due to mismatches between lab-based resistance classifications and real-world therapeutic success. Breakpoint revisions by CLSI and EUCAST, while aimed at improving safety, especially for ESBL-producing pathogens, may sometimes underestimate the efficacy of agents supported by PK/PD data (2, 3).

While this study challenges traditional CAMHB-based testing, caution is warranted against premature clinical extrapolation. RPMI-1640, though mimicking some *in vivo* conditions, lacks validation for routine use and is not standardized by CLSI or EUCAST. The observed CAZ activity under metal-depleted conditions may not hold in zinc-rich inflamed tissues, where metallo-β-lactamases like L1 could be reactivated, negating *in vitro* benefits.

Also, the study clearly demonstrates that not all antibiotics benefited equally from the medium shift. For carbapenems, unlike those shown to have reduced MIC in Enterobacterial (4), for *S. maltophila,* carbapenem MIC remains the same in both mediums. By contrast, tetracyclines showed reduced efficacy in RPMI-1640, likely due to nutrient limitations. Cefepime and aztreonam showed modest MIC reductions, less pronounced than CAZ, suggesting limited added value. These findings highlight the antibiotic-specific nature of media effects and caution against overreliance on CAZ, particularly given resistance mechanisms like TonB-dependent uptake deficiencies and efflux (5).

Moreover, the study does not assess the inoculum effect or the influence of polymicrobial infections, both highly relevant in real-world settings. *S. maltophilia* often coexists with other pathogens and produces outer membrane vesicles capable of degrading companion antibiotics, potentially confounding treatment outcomes.

We are particularly concerned about the supratherapeutic dosing used in animal models, up to 1,000 mg/kg which far exceeds human safety thresholds. Such pharmacokinetic mismatches severely limit the translatability of preclinical efficacy to clinical practice.

Given the removal of CAZ from current CLSI and IDSA treatment recommendations due to historically high resistance rates and poor clinical outcomes, its revival should be contingent upon rigorous, standardized, and clinically relevant trials (6).

We urge clinicians to maintain caution and adhere to validated susceptibility testing and regulatory guidelines until more definitive human data emerge.
